# Parenteral platforms for tunable, long-acting administration of a highly hydrophobic antiretroviral drug

**DOI:** 10.1038/s41598-024-58583-w

**Published:** 2024-05-21

**Authors:** Nima Akhavein, Marc M. Baum, Manjula Gunawardana, John A. Moss, Sandrine Calvez, Mariana Remedios-Chan, Rob Fanter, Steve Lenhard, Samantha Rusk, Leonard Azzarano, Deborah McCoy, Beat Jucker, Brian Johns, Matt Burke, Emile Velthuisen

**Affiliations:** 1 ViiV Healthcare, Five Moore Drive, Research Triangle Park, NC 27709 USA; 2 Department of Chemistry, Oak Crest Institute of Science, 128‑132 W. Chestnut Ave., Monrovia, CA 91016 USA; 3 GlaxoSmithKline, 1250 South Collegeville Road, Collegeville, PA 19426 USA; 4 Present Address: Stemline Therapeutics, 750 Lexington Avenue, 11th Floor, New York, NY 10022 USA; 5 Present Address: HemoShear, 501 Locust Ave, Charlottesville, VA 22902 USA; 6 Present Address: Janssen, 200 Great Valley Parkway, Malvern, PA 19355 USA

**Keywords:** Maturation inhibitor, Sustained release drug delivery, Long-acting HIV treatment, HIV PrEP, Ionic liquids, Subdermal implants, Drug discovery, Health care, Engineering, Materials science, Nanoscience and technology

## Abstract

GSK2838232 (GSK8232) is a second-generation maturation inhibitor (MI) developed for the treatment of HIV with excellent broad-spectrum virological profiles. The compound has demonstrated promising clinical results as an orally administered agent. Additionally, the compound’s physical and pharmacological properties present opportunities for exploitation as long-acting parenteral formulations. Despite unique design constraints including solubility and dose of GSK8232, we report on three effective tunable drug delivery strategies: active pharmaceutical ingredient (API) suspensions, ionic liquids, and subdermal implants. Promising sustained drug release profiles were achieved in rats with each approach. Additionally, we were able to tune drug release rates through a combination of passive and active strategies, broadening applicability of these formulation approaches beyond GSK8232. Taken together, this report is an important first step to advance long-acting formulation development for critical HIV medicines that do not fit the traditional profile of suitable long-acting candidates.

## Introduction

The global HIV pandemic persists as one the most severe challenges to public health and economic development, despite 40 years of progress in treatment and prevention. Approximately 1.7 million new infections worldwide occurred in 2019, with nearly two-thirds located in sub-Saharan Africa^1^. Every week, around 5500 young women aged 15–24 years become infected with HIV and are twice as likely to be living with HIV than men^1^. In the United States, HIV incidence is heterogeneous with increasing infection rates in several subpopulations^2^. The Southern region makes up 38% of the U.S. population but accounted for more than 50% of new HIV infections in 2017^3^. Certain races and ethnic groups are disproportionately affected by HIV. In 2018, Blacks/African Americans and Hispanics/Latinx accounted for approximately 39% and 16% of new HIV diagnoses, respectively^3,4^, while Black and Hispanic men who have sex with men (MSM) have experienced 4% and 14% increases in HIV infection rates, respectively^2^.

Providing at-risk individuals with long-acting biomedical options for infrequent parenteral administration of antiretroviral (ARV) agents is central to realizing improved adherence, quality of life, and extension of opportunities for prophylactic intervention. The strategy is being exploited in HIV pre-exposure prophylaxis (PrEP) using injectable and subdermal implant formulations delivering ARV drugs for four weeks, or longer^5^. In pursuit of clinical pharmacokinetic (PK) profiles that will achieve maximum efficacy and duration of action, any long-acting platform must be tunable to provide flexibility in clinical development. This is a particularly difficult barrier to overcome when delivering large doses of highly hydrophobic compounds whose absorption is mostly limited by intrinsically low solubility.

**Figure 1:**
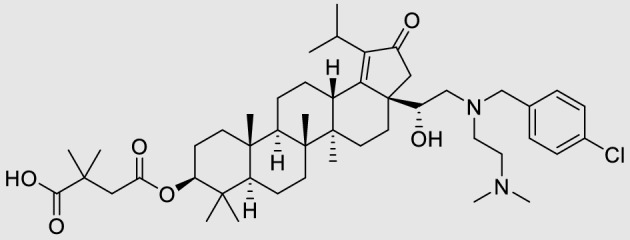
Chemical structure of GSK2838232, molecular weight 809.57.

GSK2838232 (GSK8232, Fig. 1) is a second-generation HIV maturation inhibitor (MI) that prevents the final cleavage step of the gag capsid-Sp1 polyprotein by HIV protease into the functional capsid p24, resulting in improper assembly of the virion and a noninfectious viral particle^6^.

Derived from the triterpene botulin natural product, GSK8232 exhibited an excellent virological profile against a broad panel of HIV strains, including Clade A, B, C-G viruses and isolates resistant to nucleoside reverse transcriptase inhibitors (NRTIs), non-NRTIs, and protease inhibitors (PIs)^7^. The compound demonstrated high potency against HIV-1 in cell-based assays: IC_50_ ≤ 5 nM for 97 of 101 viruses tested^7^. The in vitro IC_90_ for GSK8232 has been calculated as 6.4 nM (5 ng mL^–1^), a value that was not affected by the addition of human serum albumin even though in vitro plasma protein binding exceeded 99.9% across a range of species^8^. The IC_90_ value of 6.4 nM (termed protein-adjusted IC_90_, PA-IC_90_) was the targeted minimal threshold (*C*_*trough*_) for evaluation in human subjects. In clinical trials, GSK8232 exhibited well-defined PKs, safety, and tolerability in non-HIV infected individuals following oral administration^8–10^.

Despite the promising clinical results obtained with GSK8232 oral dosage forms, the compound’s physical and pharmacological characteristics represent opportunities for exploitation in long-acting parenteral formulations. The compound has low solubility in biorelevant media, 1 µg mL^–1^ in simulated gastric fluid (SGF, pH 1.6) and 84 µg mL^–1^ in fasted state simulated intestinal fluid (FaSSIF, pH 6.5). In preclinical studies, GSK8232 was found to have low to moderate oral bioavailability, 6–40%, depending on species and formulation^8^, that would be overcome in a parenteral device. The low clinical target *C*_*trough*_ of GSK8232 meets a key prerequisite for formulation into injectable or implantable drug delivery systems for long-term use. Here we demonstrate tunable, sustained drug release profiles of three complementary long-acting biomedical approaches.

## Materials and methods

### Chemicals

GSK8232 was kindly provided by the GSK Infectious Disease discovery unit in Research Triangle Park (RTP), NC.

### API suspension

**Figure 2:**
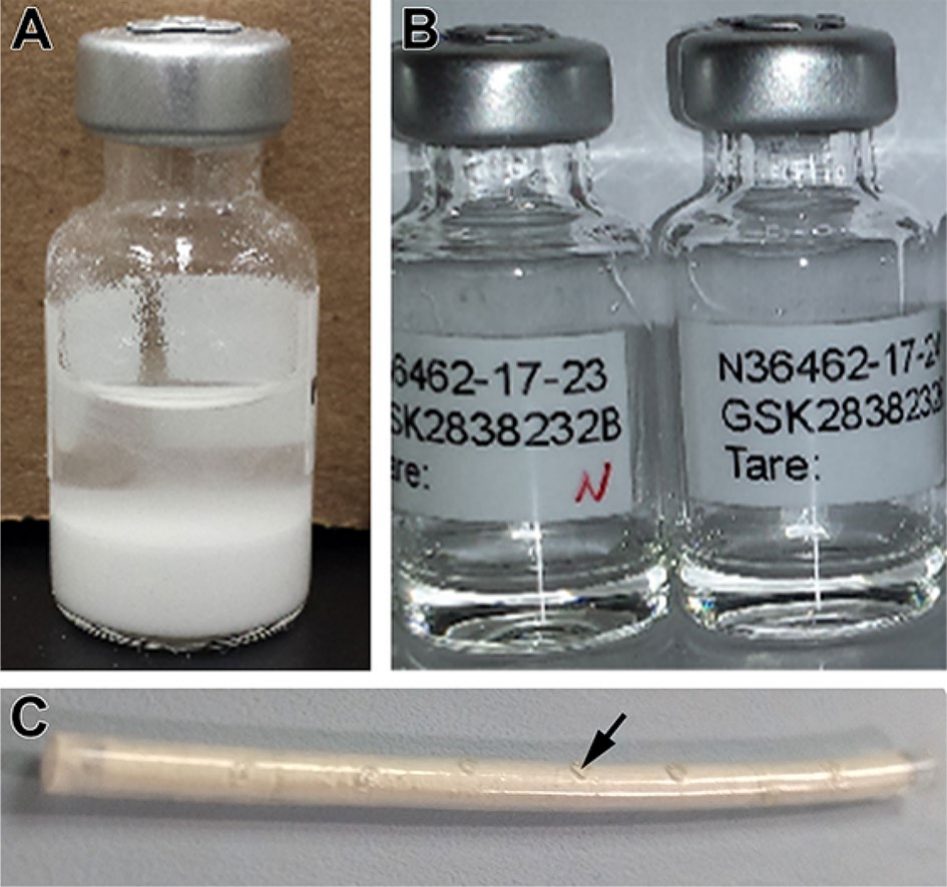
Representative photographs of GSK8232 long-acting formulations. (**A**) API suspension. (**B**) Ionic liquid. (**C**) Human-sized subdermal implant (length, 40 mm; outer diameter, 2.5 mm); arrow identifies delivery channel.

Suspensions of GSK8232 (Fig. 2A) were prepared at two different mean particle sizes determined by dynamic light scattering: 10 µm (unmilled) and 1 µm (milled). Unmilled formulations were prepared by suspending the active pharmaceutical ingredient (API) in a sterile vehicle containing Tween-80 (0.2% v/v), PEG 3350 (2% v/v), mannitol (4.5% w/v) in phosphate-buffered saline (PBS, 10 mM). Milled formulations were prepared by wet bead milling (WBM) using a MM300 mixer mill (Retsch, Germany). Briefly, unmilled suspensions were transferred into grinding chambers prefilled with YTZ grinding media 0.3 mm (Tosoh, Amsterdam, Netherlands). Formulations were milled at 25 Hz for 4 h to achieve a mean particle size of 1 µm. All formulations were sterilized by autoclaving at 121 °C for 15 min.

### Ionic liquids

GSK8232 ionic liquid (IL) lead formulations (Fig. 2B) were prepared by mixing the API and glutaric acid (counter ion) in a 2:1 molar ratio with ethanol for 2 h at room temperature (larger library screens of counter ions for GSK8232 IL formulations were carried out before glutaric acid was finally selected). Ethanol then was removed *in vacuo* by rotary evaporation to create the final composition of the IL (Table 1). In addition to visual observation of eutectic mixture formulation, ILs were also examined under optimal microscopy for birefringence. To reduce the viscosity and improve injectability of the resulting IL, polyethylene glycol 200 (PEG-200) (10% w/w) were added to the formulation.

**Table 1:** GSK8232 Ionic liquid formulation composition.

Component	Concentration (% wt/wt)	Function
GSK8232	25	Active ingredient
Glutaric acid	5	Counter-ion
PEG200	70	Viscosity reducer

### Subdermal implants

Human-sized (length, 40 mm; outer diameter, 2.5 mm, Fig. 2C) and rat-sized (length, 10–20 mm; outer diameter, 2.5 mm) GSK8232 implants were fabricated using methods described previously^11^. In the current study, aqueous solutions/suspensions (100 mg total solids) of GSK8232 with either human serum albumin (HSA) or rat serum albumin (RSA) excipients were lyophilized to produce homogeneous solid mixtures as white powders that were used to fill the silicone scaffolds.

### In vitro release studies of subdermal implants

In vitro release studies using single implants were carried out as described previously^11^, with minor modifications. Human-sized implants (exposed total surface area, 6.0 mm^2^) using HSA as an excipient (0–50% wt/wt) were incubated in PBS (pH 7.2, 100 mL) or human plasma (100 mL) containing sodium azide (0.01% wt/wt) at 37 °C and 100 RPM.

Samples were collected at predetermined timepoints for subsequent analysis by LC–MS/MS. Plasma sample purification was carried out in a 96-well format using a protein removal system (Impact, Phenomenex, Inc., Torrance, CA) according to the manufacturer’s instructions. The purified samples were dried in vacuo using a SpeedVac concentrator system (Savant SC210A Plus, Thermo Fisher Scientific, Inc.) and were reconstituted in 0.1% (vol/vol) formic acid in water (100 μL) prior to analysis. Levonorgestrel (LNG) was used as the internal standard (IS).

Sample analysis was carried out using a high-performance liquid chromatography (HPLC) system consisting of a model G1367A well-plate autosampler and a model G1312A binary pump (1200 series; Agilent Technologies, Santa Clara, CA) interfaced to an API 3000 triple-quadrupole tandem mass spectrometer (AB Sciex, Framingham, MA) with a turbo ion spray electrospray ionization (ESI) source. An Agilent Zorbax Eclipse XDB-C18 rapid resolution column (2.1 × 50 mm; 3.5 µm) controlled at 40 °C was the stationary phase. The following gradient program was used (A, 0.1%, vol/vol, formic acid in water; B, 0.1%, vol/vol, formic acid in acetonitrile): 8.0-min ramp from 100:0 A:B to 5:95 A:B, 0.1-min ramp from 5:95 A:B to 100:0 A:B, and 1.9-min hold at 100: 0 A:B. This program resulted in a total run time of 10 min with the following retention times: GSK8232, 5.7 min; LNG, 5.0 min. The measured transition ions, *m/z*, under ESI^+^ ionization mode were the following: GSK8232, 809.7 amu (parent) and 255.5 amu (product); LNG (IS), 313.1 amu (parent) and 109.3 amu (product).

### Animals

Pharmacokinetics (PKs) and preliminary safety animal studies were conducted at the GSK Upper Merion Facility (709 Swedeland Road, King of Prussia, PA). Animals were handled in strict accordance with the Guide for the Care and Use of Laboratory Animals^12^ and all experimental protocols were approved GSK U.S. Institutional Animal Care and Use Committee. Sprague–Dawley male rats (mean weight, 0.375 kg) were used throughout the study. Animals were housed under standard conditions, had ad libitum access to water and a standard laboratory diet. This study is reported in accordance with ARRIVE guidelines.

### In vivo pharmacokinetic studies

For the API suspension study, drug product was administered at 20 mg kg^–1^ dose based on animal weights measured just prior to dosing. Gastight glass syringes (100 µL volume) equipped with a 25G needle were weighed pre- and post-dose to determine the amount of GSK8232 injected into the right gastrocnemius muscle of the Sprague Dawley rats. Blood samples for PK analysis were collected at predetermined timepoints [Day 0 (0 (pre-dose), 60, 120, 240 min), Days 1, 2, 4, 7, 14, 21, 28] via a lateral tail vein snip into tubes containing K_2_EDTA. Whole blood samples were frozen on dry ice immediately and stored at − 80 °C.

For the IL study, drug product was administered at 20 mg kg^–1^ based on animal weights measured just prior to dosing. Gastight glass syringes (50 or 100 μL) equipped with 25G needles were weighed pre- and post-dose to determine amount of GSK2838232 administered. Injection sites were shaved immediately prior to dosing and the sites observed immediately after the needle was withdrawn to note any dose leakage. Dose was injected IM into the right gastrocnemius muscle. Blood samples for PK analysis were collected at predetermined timepoints [Day 0 (0 (pre-dose), 60, 120, 240, 480 min), Days 1, 2, 4, 7, 14, 21, 28] via a lateral tail vein or tail tip amputation into tubes containing K_2_EDTA. Whole blood samples were frozen on dry ice immediately and stored at − 80 °C.

For the implant study, rats were momentarily anesthetized with isoflurane inhalant (1–5%) and the right flank was shaved, wiped with alcohol, and then allowed to dry before dosing. A small incision was made in the skin on the right flank with a #10 scalpel. GSK8232-loaded implants were inserted with either a sterile 6G needle assembly or with sterile forceps. Following implant insertion, the skin was closed with Vetbond sterile glue. Blood samples for PK analysis were collected at predetermined timepoints (vide infra) via a lateral tail vein or tail tip amputation into tubes containing K_2_EDTA. Whole blood samples were frozen on dry ice immediately and stored at − 80 °C.

### Bioanalysis

The GSK8232 drug substance was extracted from rat blood (20 μL) by protein precipitation using acetonitrile containing formic acid (0.1% vol/vol) and isotopically labelled internal standard (IS), [^2^H_7_]‑GSK8232. Sample tubes were capped and vortex-mixed thoroughly for approximately 5 min, followed by centrifugation for at least 5 min at approximately 3000 × g. Then, a supernatant aliquot (50 µL) was transferred into clean tubes containing acetonitrile/water/formic acid (50/50/0.1, vol/vol/vol, 150 µL). The resulting samples were mixed by vortex agitation for approximately 2 min and were analyzed by UPLC–MS/MS.

Chromatographic separation of GSK8232 and the IS from other matrix components was performed using an ACQUITY UPLC on a model BEHC18 (Waters Co., Milford, MA) column (50 mm × 2.1 mm, i.d. 1.7 μm), controlled at 40 °C during the analysis. The mobile phases consisted of: A, ammonium formate (1 mM) with formic acid (100/0.05, vol/vol); B, acetonitrile with formic acid (100/0.1, vol/vol). Isocratic liquid chromatographic conditions (63% B) were used at a flow-rate of 0.6 mL min^-1^. The typical injection volume was 5 µL in a 100 µL sample syringe.

An AB Sciex API-5000 (Framingham, MA) equipped with a TurboIonspray interface (TIS) was operated in the positive ionization mode. Quantification was carried out on a multiple reaction monitoring (MRM) and ion transitions of *m/z* 809 → 255 and 816 → 262 were chosen for GSK8232 and [^2^H_7_]-GSK8232, respectively. The following optimized mass spectrometric conditions were used for GSK8232 and the IS: TIS source temperature, 600 °C; TIS voltage, 5500 V; curtain gas, 20 psi (nitrogen); nebulizing gas (GS1), 75 psi (zero air); TIS gas (GS2), 75 psi (zero air); collision energy, 50 eV; declustering potential, 80 eV. The lower limit of quantification (LLQ) for GSK8232 was 0.5 ng mL^–1^ in a 20 μL aliquot of rat blood.

### Magnetic resonance imaging

Magnetic resonance imaging (MRI) was performed in order to temporally assess the drug depot kinetics and inflammatory infiltrate on a 4.7 Tesla Bruker magnet (Bruker Biospin GmbH, Germany) equipped with an 11.6 cm diameter actively shielded gradient set (400 mT m^–1^). A birdcage volume coil (Bruker Biospin GmbH) with an inner diameter of 72 mm was used to obtain optimal radiofrequency homogeneity over the volume of interest. Rats were anesthetized with continuously inhaled isoflurane (1.5–2%) and a constant body temperature of 37 °C was maintained using a circulating water heating blanket. Respiration was monitored using a respiratory sensor (SA Instruments, Inc., Stony Brook, NY) placed on the abdomen of the animal.

Following a tri-pilot gradient-echo image, a T_2_-weighted RARE (Rapid Acquisition with Relaxation Enhancement) imaging sequence with fat suppression was used to acquire scans consisting of coronal slices (10 slices per scan) to cover the entire drug depot using the following parameters: TR/TE, 2000/20 ms (effective *T*_*2*_ = 60 ms); RARE Factor (number of echo), 8; bandwidth, 50 kHz; field of view, 10 × 10 cm; matrix, 128 × 128; slice thickness, 2 mm, number of averages, 8; and acquisition time, 4 min 16 s. Following the coronal scan a series of axial scans (10 slices per scan) were acquired using the following parameters: TR/TE, 2000/20 ms (effective *T*_*2*_ = 60 ms); RARE Factor (number of echo), 8; bandwidth, 50 kHz; field of view, 7 × 7 cm; matrix, 128 × 128; resolution, 547 µm; slice thickness, 2 mm; number of averages, 8; and acquisition time, 4 min 16 s.

Ultra-small paramagnetic iron oxide (USPIO) MRI was performed to image macrophage infiltration into the drug depot region. The USPIO contrast uptake in the muscle was measured using a *T*_2_^*^-weighted imaging MGE (Multiple Gradient Echo) sequence acquiring 1 to 3 adjacent scans consisting of axial slices (10 slices per scan) to cover the entire drug depot using the following parameters: TR/TE, 750/2.3 ms; #echoes, 10; echo spacing, 6 ms (2.34–56.34 ms); bandwidth, 75 kHz; flip angle (FA), 30°; field of view, 7 × 7 cm; matrix, 128 × 128; resolution, 547 µm; slice thickness, 2 mm; number of averages, 1; and acquisition time, 1 min 36 s. MRI image analysis of drug depot volume was performed using Jim 8 analysis software (Xinapse Systems, Inc, UK).

## Results

### Pharmacokinetics and magnetic resonance imaging of GSK8232 suspensions

**Figure 3:**
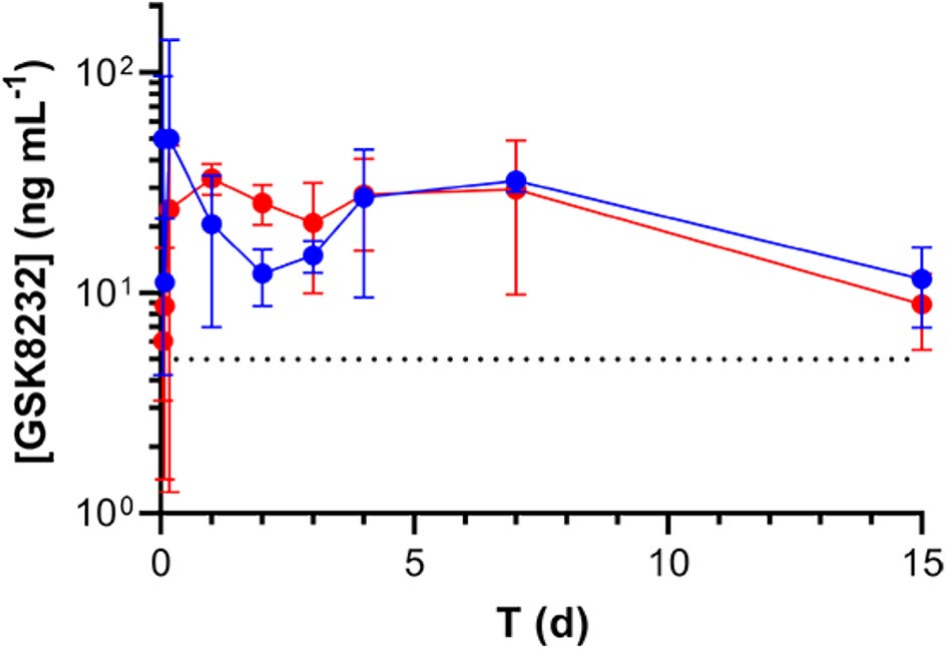
Concentration–time profiles of unmilled (10 µm particle size, blue) and milled (1 µm particle size, red) GSK8232 suspensions in rats. Each circle represents mean ± *SD* (*N* = 4 animals per group); broken horizontal line represents the PA-IC_90_ (5 ng mL^–1^).

Pharmacokinetic profiles of GSK8232 API suspensions were evaluated in rats over 15 days (Fig. 3). Intramuscular administration of both 1 µm and 10 µm suspensions resulted in sustained blood levels over PA-IC_90_ for the entire 15-day period. There was no apparent difference in the PK profiles as a result of particle size.

**Figure 4:**
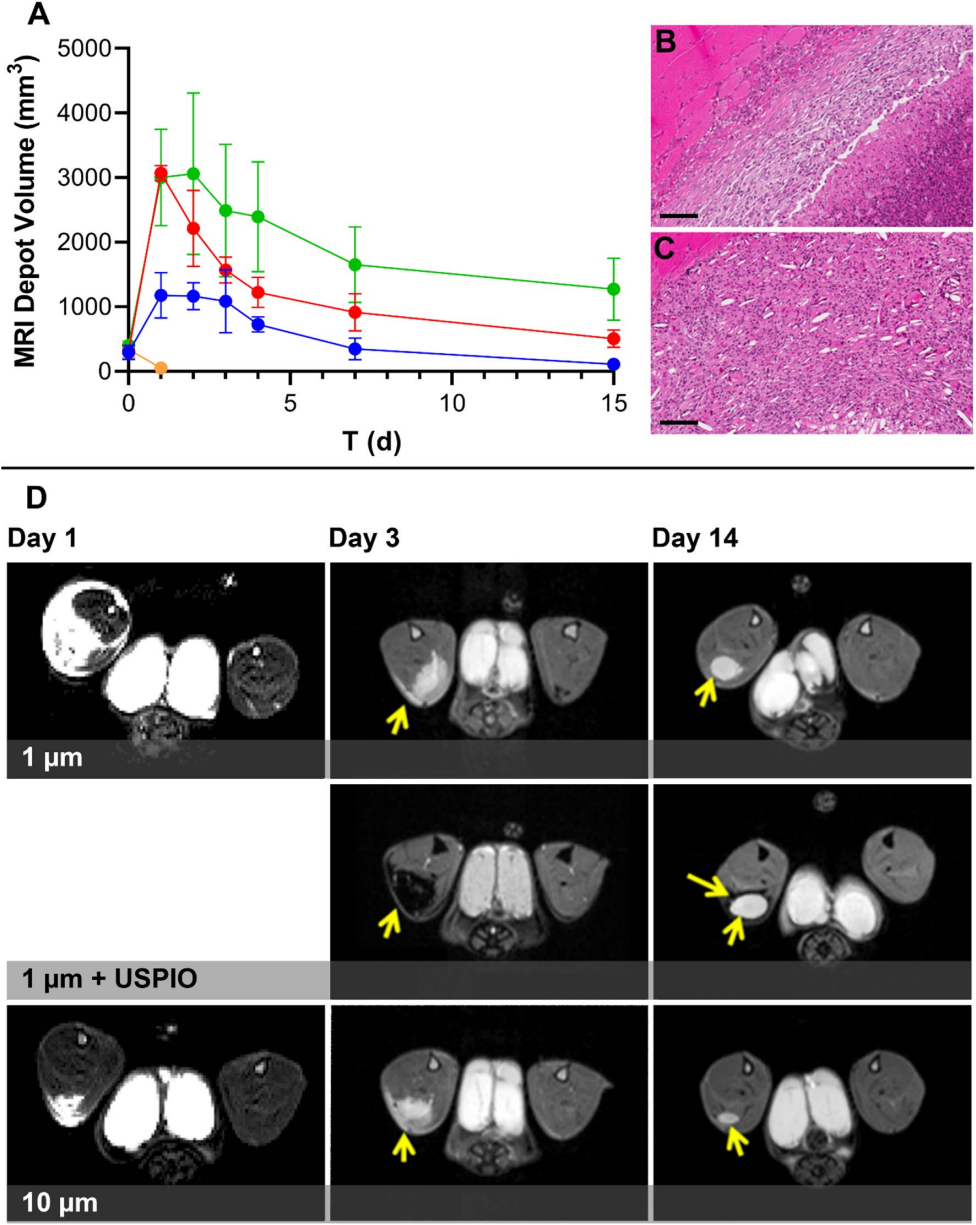
Depot volume kinetics, histology, and MRI analysis of GSK8232 suspensions in rats. (**A**) MRI T2 weighted depot volumes timecourse over 15 days post injection. Each circle represents mean ± *SD* (*N* = 4 for medicated groups); blue, unmilled (10 µm particle size); red, milled (1 µm particle size); green, 1 µm particle size and USPIO; orange, vehicle control. (**B**) H&E stained depot region of the muscle tissue section from Animal 7, 1 µm particle group collected at 20 × magnification. Scale bar, 100 µm. (**C**) H&E stained depot region of the muscle tissue section from Animal 9, 10 µm particle group collected at 20 × magnification. Scale bar, 100 µm. (**D**) Magnetic resonance images are shown for Days 1, 3, and 14 (columns) for the following formulations (rows): 1 µm group (top row); 1 µm + paramagnetic iron oxide (USPIO) MRI contrast agent group (center row), and 10 µm group (bottom row). The arrowheads identify the depot, while in the 1 µm + USPIO group the arrowhead identifies the iron-laden depot on Day 3; the iron (top arrow) and bright depot (bottom arrow) on Day 14.

T_2_-weighted RARE MRI was used to assess the sub-acute inflammatory response resulting from GSK8232 suspensions in rats. A threefold increase in depot volume in the 1 µm particle size group, compared to the 10 µm group, was observed at Day 1 post injection. Representative images from an animal in each group are included below to highlight the difference in inflammatory volume across both groups (Fig. 4D). Representative animals at Day 14 highlight the difference in depot volume between the 1 µm and 10 µm groups (Fig. 4D). Overall, there was no qualitative relationship between changes in depot volume over time (Fig. 4A) and corresponding GSK8232 plasma concentrations (Fig. 3). Interestingly, the observed difference in early depot volume at Day 1 post injection did not correspond to the plasma peak drug concentration at the same timepoint. However, the 1 µm particle size group did exhibit an increased plasma peak drug concentration at 24 h post injection relative to the larger, 10 µm particle size group. This increased peak concentration is in many ways expected and likely due faster dissolution rate, and thus faster absorption rate of the smaller particles due to increased surface area. This trend was maintained out to 3 d post injection and, plasma peak drug concentrations were similar between the two groups at Day 4. Twenty four h post USPIO injection, the entire depot region was hypointense as a result of uptake of this negative MRI contrast agent (Fig. 4D), but by Day 14 only the outside perimeter of the depot was hypointense to reflect the macrophage encapsulation region of the depot. Histological evaluation confirmed the USPIO was co-localized with macrophages around the perimeter of the depot (vide infra).

The early depot volume differences observed between the two different particle size groups translated to marked histological differences. Histological examination of the hindlimb muscle in the 10 µm group (Fig. 4C) revealed the following observations: moderate foamy macrophage infiltration; multinucleated giant cells; and minimal to mild necrosis with inflammatory cell infiltrate. However, in the 1 µm group (Fig. 4B), marked necrosis/inflammatory cell infiltrate with granular/eosinophilic materials, nuclear debris, neutrophils, and macrophages were observed in the H&E-stained tissue sections. While it is interesting that these differences in depot volume did not translate to significantly different overall PK profiles, this observation does suggest particle size is a contributing factor to the overall tolerability an intramuscularly-administered formulation.

### Rat pharmacokinetic profiles of GSK8232 ionic liquid formulations

Ionic liquid formulations of GSK8232 were evaluated in rat PK studies spanning one month. Preliminary neat ILs were highly viscous, necessitating additional formulation excipients to be added as described above to improve injectability.

**Figure 5:**
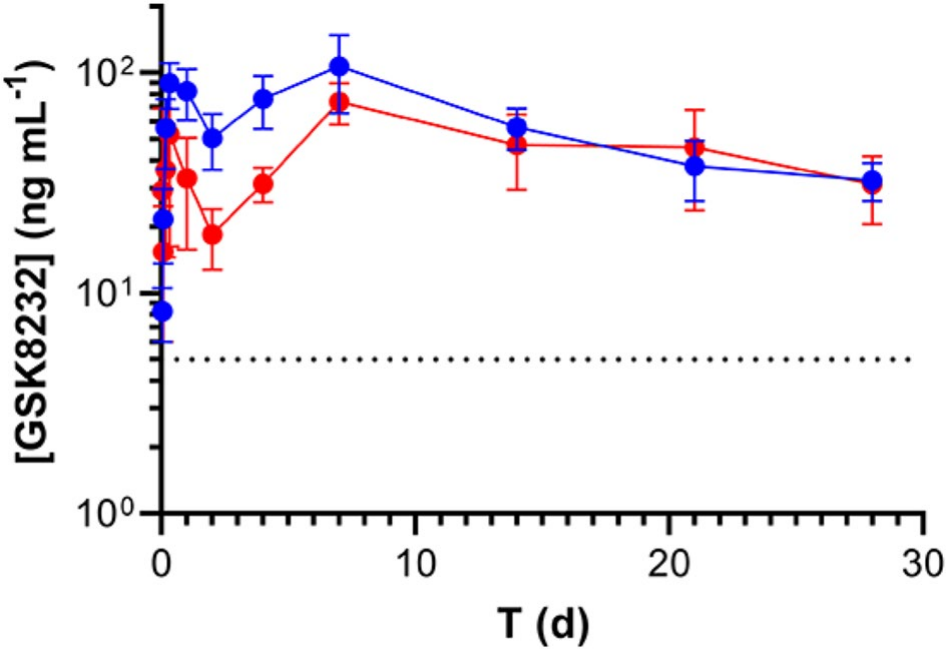
Concentration–time profiles in rats following subcutaneous injection of GSK8232 (dose 20 mg kg^–1^) ionic liquid formulations using glutaric acid as the counter ion (Table 1) at 150 mg mL^–1^ (blue) and 182 mg mL^–1^ (red) GSK8232 concentrations. Each circle represents mean ± *SD* (*N* = 3 animals per group); broken horizontal line represents the PA-IC_90_ (5 ng mL^–1^).

Two lead formulations were injected intramuscularly at a dose of 20 mg kg^–1^ and the corresponding PK profiles are shown in Fig. 5. The primary difference between the formulations was the API concentration (see Fig. 5 legend).

### In vitro GSK8232 release from subdermal implants

**Figure 6:**
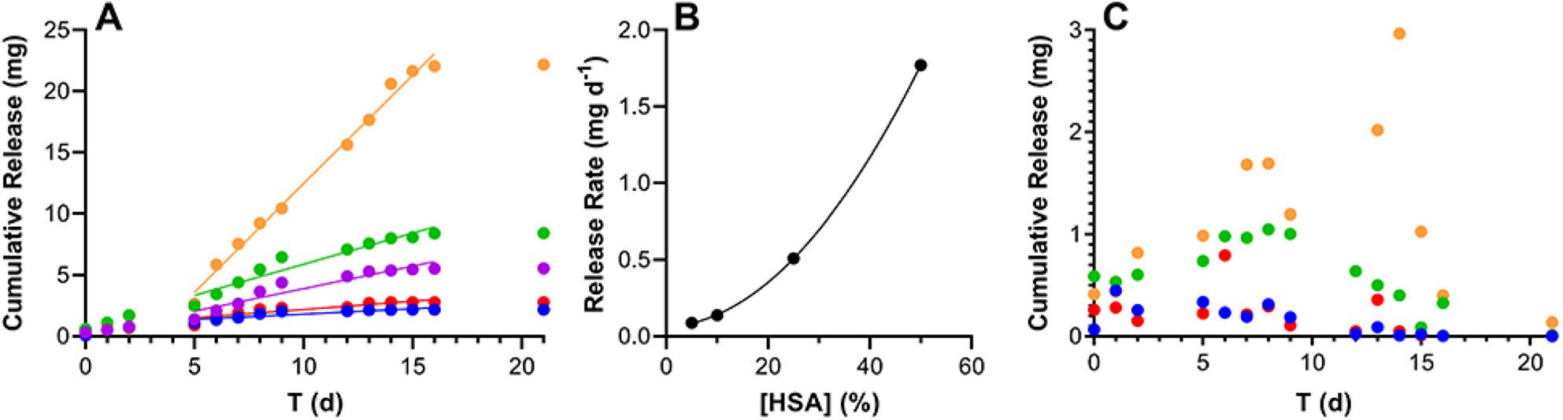
In vitro release profiles of human-sized (40 mm long; 6.0 mm^2^ exposed drug-permeable surface area) subdermal GSK8232 implants co-formulated with HSA as the only excipient. Blue circles, 5% wt/wt HSA; red circles, 10% wt/wt HSA; green circles, 25% wt/wt HSA; orange circles, 50% wt/wt HSA. (**A**) Cumulative GSK8232 release into PBS. Solid lines correspond to linear regressions between 5 and 16 days. Magenta circles, 100% GSK8232. (**B**) Second order polynomial (quadratic) least squares fit of GSK8232 release rate, calculated from the slopes of the linear regressions in (**A**), and the HSA content (*y* = 0.0610 + 0.00150*x* + 0.000654*x*^*2*^; *R*^*2*^ = 1.000). (**C**) Cumulative GSK8232 release into human plasma.

In vitro cumulative release profiles of GSK8232-HSA implant prototypes exhibited burst-free, sustained release with zero-order (linear) kinetics over 21 days (Fig. 6A). The release rate correlated with the amount of HSA excipient in the implant (Fig. 6B), with the highest release rate observed with 50% wt/wt HSA (Table 2). In vitro release into human plasma led to low detectable drug concentrations and noisy data (Fig. 6C), likely due to the high protein binding (> 99.9%) of GSK8232^8^. A small amount of cumulative drug release was observed over the first 15 d for HSA implant concentrations of 25 and 50% wt/wt (Table 2).

**Table 2:** Comparison of GSK8232 subdermal implant cumulative in vitro release rates into PBS and human plasma as a function of HSA content.

[HSA] (% wt/wt)	In vitro release rate (mg d^–1^), *R*^2^	
	PBS^a^	Human plasma
0	0.37, 0.9013	–
5	0.088, 0.8050	N/A
10	0.14, 0.8124	N/A
25	0.51, 0.9262	0.065^b^, 0.9076
50	1.77, 0.9912	0.15^c^, 0.8630

**Figure 7:**
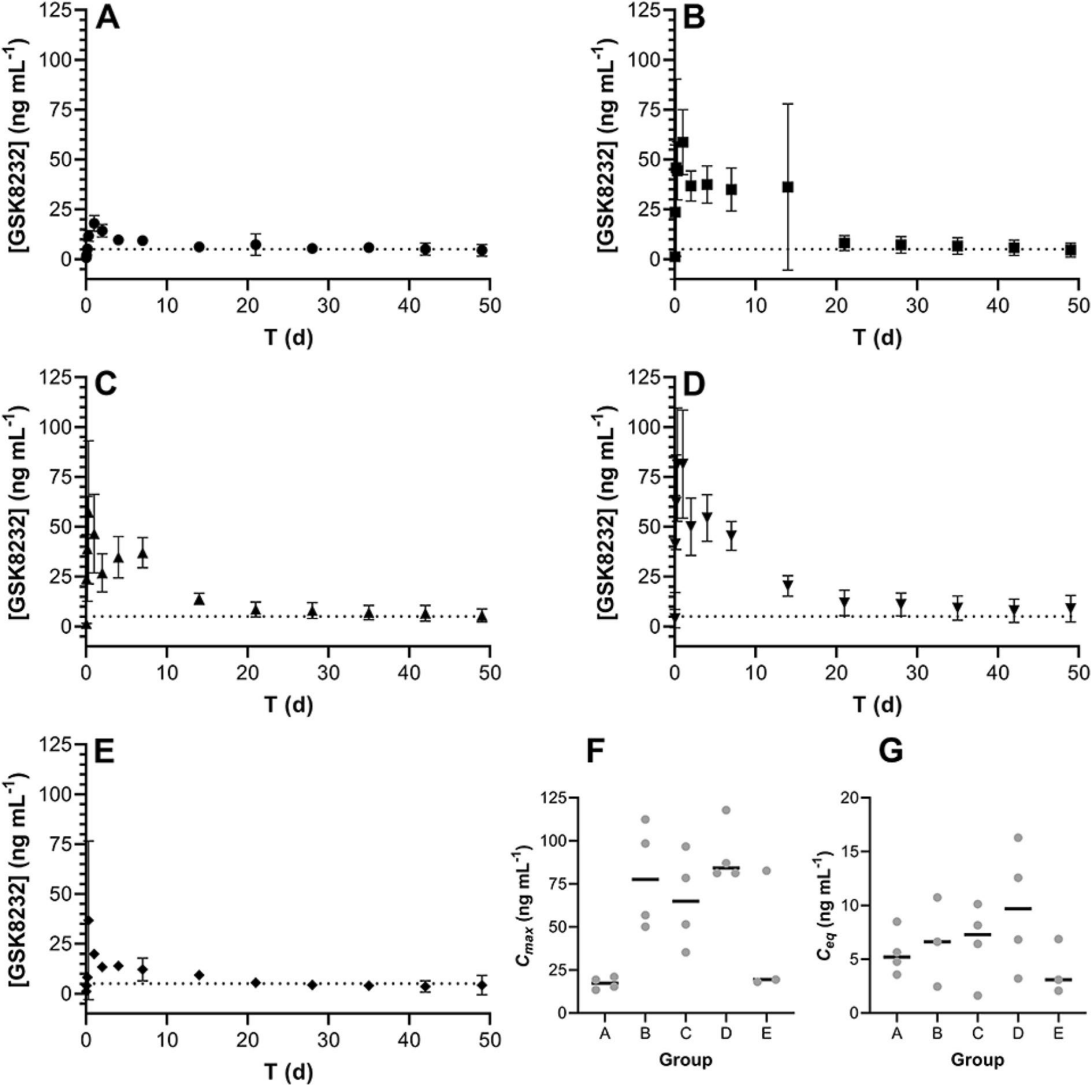
Subdermal implantation of GSK8232 subdermal implants maintained sustained drug levels in rats over 49 days; broken line, GSK8232 in vitro PA-IC_90_ (5 ng mL^–1^). (**A**) Group A: 100% GSK8232 (0% wt/wt RSA); exposed drug-permeable surface area, 3.4 mm^2^; length, 10 mm; *N* = 4; (**B**) Group B: 90% wt/wt GSK8232 (10% wt/wt RSA); exposed drug-permeable surface area, 3.4 mm^2^; length, 10 mm; *N* = 4; (**C**) Group A: 75% wt/wt GSK8232 (25% wt/wt RSA); exposed drug-permeable surface area, 3.4 mm^2^; length, 10 mm; *N* = 4; (**D**) Group D: 50% wt/wt GSK8232 (50% wt/wt RSA); exposed drug-permeable surface area, 3.4 mm; length, 20 mm^2^; *N* = 4; (**E**) Group E: 90% wt/wt GSK8232 (10% wt/wt RSA); exposed drug-permeable surface area, 1.7 mm^2^; length, 10 mm; *N* = 3; (**F**) *C*_*max*_ values for all study groups; black lines, medians; grey circles, individual values; (**G**) equilibrium concentration (median D21-49) *C*_*eq*_ values for all study groups; black lines, medians; grey circles, individual values.

A release rate into human plasma could not be calculated for 5–10% w/w HSA (see Fig. 7C).

^a^Day 5–16, ^b^Day 0–8, ^c^Day 0–14.

### Rat pharmacokinetic profiles of GSK8232 subdermal implants

GSK8232 implants co-formulated with RSA as the sole excipient (drug content, ca. 7.5 mg for all groups), based on the in vitro results shown in Fig. 6, were evaluated in rats (five study groups, 3–4 animals per group) over 49 days and the results are summarized in Fig. 7.

The implants exhibited a small, initial burst release (note linear *y*-axis scale) and maintained stable plasma drug concentrations for the 49 d dosing period. In all cases, except for Group E, the implants maintained GSK8232 plasma exposure above the protein-adjusted-IC_90_ which is the minimum plasma concentration target for efficacy. While *C*_*max*_ values did not correlate with implant RSA content (Fig. 7F), median equilibrium drug concentrations, *C*_*eq*_, did increase with RSA content for implants with the same total exposed surface area (Fig. 7G), as expected based on in vitro release data (Fig. 6B).

## Discussion

Maturation inhibitors represent a potentially new mechanism of action for the treatment and prevention of HIV infection. The unique physicochemical and antiviral properties of the maturation inhibitor GSK8232 provided an interesting opportunity to explore a variety of parenteral delivery platforms that could be used for HIV PrEP.

In this report, we pursued long-acting formulations of a hydrophobic drug with relatively large predicted dose requirements. The combination of dose and drug solubility presents unique design constraints and requires careful balance of excipient selection, drug loading, and tunability of drug release rates. With these constraints in mind, three formulation approaches were engineered and screened for their potential to yield sustained, meaningful systemic concentrations of GSK8232 in preclinical models. Although the formulations presented here were customized for GSK8232, our goal was to identify up to three general approaches that could be adapted to other compounds with similar properties and challenges (e.g. integrase inhibitors).

To take full advantage of the low aqueous solubility of GSK8232, simple API suspensions were first formulated. Blood drug concentrations were sustained for *ca*. 2 weeks over the estimated PA-IC_90_ after intramuscular administration in rats. While particle size did not seem to have a significant impact on the PK profiles (Fig. 3), there were differences in measured depot volumes observed by MRI (Fig. 4). Specifically, IM injections of smaller particles resulted larger depot volume over a 24-h period. Together with histological analyses, these data suggest that particle size does have an impact on sub-acute inflammatory response that could potentially lead to longer-term differences in PK profiles and injection site tolerability.

Ionic liquid formulations subsequently were evaluated as a long-acting approach for GSK8232. While ILs have a wide range of applications in the pharmaceutical industry and beyond, ours is the first report that demonstrates their use as long-acting injectable formulations. The advantage of the GSK8232 IL systems is that the drug is in solution (i.e. not a suspension) in the final formulation, there-by circumventing any potential development risks due to particle settling over time. A solution formulation also mitigates long-term physical stability risks (e.g. Ostwald ripening). Formulations can also be prepared at higher API concentration without any concern for syringe compatibility due to particle aggregation which allows for larger doses to be administered without increasing injection volumes. This is especially important in reducing dosing frequency of long-acting formulations.

The final long-acting formulation approach employed for GSK8232 in this report was an innovative subdermal implant reservoir design, previously used for water-soluble drugs^11,13^. The implant was engineered to exploit a combination of delivery channels in the outer implant shell and solubility enhancers as part of the formulation to ultimately tune the API release rate. This design gives the formulator more tools to dial-in a release rate that will yield clinically relevant PK profiles. In our specific case and because of the low aqueous solubility of GSK8232, diffusion-mediated release is generally expected to be slow and, in many ways, the challenge of this drug delivery format was to produce an accelerated release rate while maintaining an overall sustained PK profile We demonstrate in this report that clinically meaningful drug release rates of GSK8232 are achieved using this approach. While burst release control was not a major issue in our case, it could inevitably be for a faster-releasing compound. In those cases, burst release could be controlled by removing solubility enhancers like serum albumin and by reducing the overall area of the delivery channels. This reservoir design ultimately gives the formulator a number of ways to dial-in a specific release rate that will yield clinically-meaningful pharmacokinetic profiles.

Formulation innovation will be critical to enable more patient-centric medicines for HIV patients around the world. The additional barriers of large dosing volume requirements along with two or three-drug combinations has made long-acting formulation development particularly challenging for HIV treatment. A combination of passive and active drug delivery strategies was effectively applied in this report to develop long-acting formulations of GSK8232. The future success and design of these platforms certainly will depend on other manufacturing and product performance considerations including scaleup requirements and viscosity. Long-term stability and safety for any new formulations will also be an important challenge for any clinical program. Furthermore, dosing internals will need to be carefully designed to minimize exposures under the PAIC90 which could overtime lead to resistance. Such dosing strategies are developed through clinical development programs and are an important challenge in developing long-acting HIV medicines. However, in this report, we take important first steps to advance long-acting formulation development for these important medicines.

## Conclusion

The PKs and preliminary safety of three innovative, long-acting parenteral drug delivery platforms are compared using the HIV maturation inhibitor GSK8232 as the API. All three approaches led to sustained GSK8232 plasma exposure above the therapeutic target for periods spanning 15 d (injectable suspensions), 30 d (ILs), and 49 d (subdermal implants). The PK profiles were widely tunable despite formulation challenges related to the highly insoluble agent.

Our study constitutes an important step in developing a flexible toolkit for long-acting parenteral ARV delivery, as these products advance through preclinical development in pursuit of eventual, optimal clinical profiles for HIV prevention and treatment.

## Data Availability

All other data supporting the findings of this manuscript are available from the corresponding author (NA) upon reasonable request.
